# Association between coronal caries and malocclusion in an adult population

**DOI:** 10.1007/s00056-020-00271-1

**Published:** 2020-12-18

**Authors:** Olaf Bernhardt, Karl-Friedrich Krey, Amro Daboul, Henry Völzke, Christian Splieth, Thomas Kocher, Christian Schwahn

**Affiliations:** 1 Department of Restorative Dentistry, Periodontology, Endodontology, Preventive Dentistry and Pediatric Dentistry, University Medicine Greifswald, Walther-Rathenau-Str. 42a, 17475 Greifswald, Germany; 2 Department of Orthodontics, University Medicine Greifswald, Walther-Rathenau-Str. 42a, 17475 Greifswald, Germany; 3 Department of Prosthetic Dentistry, Gerodontology and Biomaterials, University Medicine Greifswald, Walther-Rathenau-Str. 42a, 17475 Greifswald, Germany; 4 Institute for Community Medicine, University Medicine Greifswald, Walther-Rathenau-Str. 48, 17475 Greifswald, Germany

**Keywords:** Dental occlusion, Orthodontics, Caries risk assessment, Epidemiology, Multilevel analysis, Okklusion, Kieferorthopädie, Kariesrisikobewertung, Epidemiologie, Mehrebenenanalyse

## Abstract

**Purpose:**

Only a few but conflicting results have been reported on the association between malocclusions and caries. We investigated this association using data from the population-based cross-sectional Study of Health in Pomerania (SHIP).

**Methods:**

Sagittal, vertical and transversal intermaxillary relationship, space conditions and sociodemographic parameters of 1210 dentate subjects (median age 30 years, interquartile range 25–35 years) were collected. Caries was assessed with the Decayed-Missing-Filled Surfaces index but analyzed as ordered outcome (four levels: sound, enamel caries, caries, tooth loss) in ordinal multilevel models, taking into account subject, jaw, and tooth level simultaneously.

**Results:**

Anterior open bite ≤3 mm (odds ratio [OR] = 2.08, 95% confidence interval [CI]: 1.19–3.61), increased sagittal overjet of 4–6 mm (OR = 1.31, CI: 1.05–1.64), distal occlusion of ½ premolar width (OR = 1.27, CI: 1.05–1.53) and distal 1 premolar width (OR = 1.31, CI: 1.06–1.63) were associated with adjusted increased odds for a higher outcome level (caries). Anterior spacing (OR = 0.24, CI: 0.17–0.33), posterior spacing, (OR = 0.69, CI: 0.5–0.95), posterior crowding (OR = 0.57, CI: 0.49–0.66) and buccal nonocclusion (OR = 0.54, CI: 0.33–0.87) were associated with a lower outcome level (caries).

**Conclusion:**

The results from this population-based study suggest that a connection between caries and malocclusion exists to a limited extent in young adults. The associations with caries are contradictory for several malocclusion variables. Distal occlusion (OR = 1.31, CI: 1.06–1.63) and related skeletal anomalies displayed positive associations with caries whereas crowding did not. Orthodontic treatment of anterior crowding would probably not interfere with caries experience. These aspects should be considered for patient information and in treatment decisions.

## Abstract (de)

**Ziel:**

Da über den Zusammenhang zwischen Malokklusionen und Karies bislang nur wenige und widersprüchliche Ergebnisse bekannt sind, haben wir diese Fragestellung anhand von Daten aus der bevölkerungsbezogenen Querschnittsstudie „Study of Health in Pomerania“ (SHIP) untersucht.

**Methoden:**

Die sagittale intermaxilläre Kieferrelation, Variablen der Malokklusion und soziodemographische Parameter von 1210 Probanden (Durchschnittsalter 30 Jahre, Interquartilbereich 25–35) wurden in die Analyse einbezogen. Karies wurde mit dem DMFS(„Decayed-Missing-Filled Surfaces“)-Index erfasst und als geordnetes Ereignis (4 Stufen: gesund, Schmelzkaries, Karies, Zahnverlust) in ordinalen Mehrebenenmodellen unter gleichzeitiger Berücksichtigung von Personen‑, Kiefer- und Zahnebene analysiert.

**Ergebnisse:**

Anterior offener Biss ≤3 mm (Odds Ratio [OR] = 2,08, 95 %-Konfidenzintervall [KI]: 1,19–3,61), vergrößerte sagittale Stufe von 4–6 mm (OR = 1,31, KI: 1,05–1,64), distale Okklusion von einer halben Prämolarenbreite (OR = 1,27, KI: 1,05–1,53) und distale Okklusion von 1 Prämolarbreite (OR = 1,31, KI: 1,06–1,63) waren mit einem erhöhten Risiko für Karies assoziiert. Lückige Zahnstellung in der Front (OR = 0,24, KI: 0,17–0,33), lückige Zahnstellung im posterioren Bereich (OR = 0,69, KI: 0,5–0,95), Engstand im posterioren Bereich (OR = 0,57, KI: 0,49–0,66) und bukkale Non-Okklusion (OR = 0,54, KI: 0,33–0,87) waren mit einem geringeren Risiko für Karies assoziiert.

**Schlussfolgerungen:**

Die Ergebnisse dieser bevölkerungsbezogenen Studie legen nahe, dass bei jungen Erwachsenen in begrenztem Umfang ein Zusammenhang zwischen Karies und Malokklusion besteht. Die Assoziationen mit Karies sind bei den einzelnen Malokklusionsvariablen nicht gleichgerichtet. Die distale Okklusion (OR = 1,31, KI: 1,06–1,63) und verwandte skelettale Anomalien zeigten positive Assoziationen mit Karies, während dies beim Engstand nicht der Fall war. Eine kieferorthopädische Behandlung des frontalen Engstandes würde das Kariesrisiko wahrscheinlich nicht beeinflussen. Diese Aspekte sollten bei der Patienteninformation und bei Behandlungsentscheidungen berücksichtigt werden.

## Introduction

Proper alignment and function of teeth as well as neutral occlusion are primary goals of orthodontic treatment. This should lead to an appealing aesthetic appearance, ensure chewing efficiency and last but not least, has been proposed to be associated with periodontal and dental health [3]. There has been a long-lasting debate about the impact of malocclusion on the progression of caries and periodontal disease [23, 24]. Although an association between malocclusion and periodontitis was established and does not seem to be negligible [4], periodontal health after orthodontic treatment does not seem to improve [6]. Regarding caries, the association with malocclusion seems to be even smaller [12].

Conflicting data have been reported in the past as to whether dental crowding increases caries scores [20]. Some studies reported higher interproximal caries prevalence, whereas others did not. Most of the studies focused on anterior teeth [2]. Differences in caries risk were also found between the upper and lower jaws [20]. Regarding caries, results from intervention studies are also heterogeneous [5, 12]. A recent publication on caries prevalence and former orthodontic treatment on 448 Australians at the age of 30 years did not provide any measurable benefits from orthodontic treatment with respect to improved dental health later in life [12]. No distinction was made in that study, however, for different forms of malocclusion, which was established with the Dental Aesthetic Index and orthodontic treatment had been performed mainly to resolve aesthetic problems [12]. In a retrospective German evaluation, 75 former Angle class II patients seemed to benefit from orthodontic treatment based on Decayed-Missing-Filled Surfaces (DMFS) values when compared to a population-based age cohort [5].

In contrast to crowding, much less is known about the relationship between overjet, overbite, crossbite, and spacing to caries. Studies in primary and mixed dentitions delivered inconsistent results [15, 19, 35, 40]. In an early study, Helm and Petersen considered different forms of malocclusion but did not find any association with caries prevalence in an adult sample [23].

To the best of our knowledge, there are no epidemiological data on the association between caries and the different forms of malocclusion including sagittal intermaxillary relationships in an adult population. Thus, we aimed to analyze cross-sectional data from the Study of Health of Pomerania (SHIP) to assess the association between caries prevalence and various forms of malocclusion in a statistical model on tooth, jaw and subject levels.

## Materials and methods

### Study participants

The aim of the population-based SHIP was to estimate the prevalence of a broad range of diseases, risk factors, and health-related factors for the Northeast German population. The baseline examination SHIP‑0, whose sampling method was adopted from the World Health Organization MONICA (Monitoring Trends and Determinants in Cardiovascular Disease) Project in Augsburg, Germany, was approved by the local ethics committee and performed between 1997 and 2001 [28]. The net sample (without migrated or deceased subjects) comprised 6265 subjects with an age range from 20 to 79 years. Finally, 4308 subjects—all were Caucasian—gave written, informed consent and participated in SHIP‑0, which corresponded to a response rate of 68.8%. SHIP‑0 comprised a medical examination, a clinical dental examination (including periodontal, orthodontic, functional, and cariologic components), an interview, and a questionnaire completed by each participant [26, 28].

### Assessment of malocclusion

The occlusal status was assessed according to selected occlusal parameters including the sagittal intermaxillary relationship in the canine region. This relationship was registered separately for the right and left canine regions and determined as neutral, distal by the width of ½ premolar and 1 premolar, and mesial by at least a ½ premolar width [25]. The following signs were recorded as being either present or absent: frontal and lateral crowding, ectopic position of canines, widely spaced teeth without approximal tooth contact, frontal and lateral crossbite, buccal nonocclusion, excessive overjet and overbite, edge-to-edge bite, open bite, negative overjet and retruded position of maxillary incisors. Orthodontic status was not recordable when in 2 or more sextants of the dentition (2 anterior and 4 posterior tooth regions), 3 or more teeth per sextant were missing, regardless of whether the gaps were restored or not. Third molars were not included in the evaluation.

### Dental examination

According to the WHO recommendations [42], coronal caries findings (cavitated carious defects into the enamel and dentine), fillings, secondary caries on the surface level, and missing teeth, were registered by surface with the exception of third molars according to the half-mouth method (quadrants 1 and 4, or quadrants 2 and 3 in alternating sequence) using a periodontal probe (PCP 11, Hu Friedy, Frankfurt am Main, Germany) [26, 39]. Cavitated carious lesions (D component) were subdivided into lesions confined to enamel and those involving dentine. The number of cavitated lesion solely in enamel was absolutely minimal (*n* = 72). Initial caries lesions without cavitation were not recorded or counted for the caries scores. In detail, caries was defined in the manual of SHIP‑0 as follows:

0. Sound: no caries, discoloration without carious defect, wedge-shaped defects, fissure sealings, tooth brushing defects
1. Enamel caries or carious defect into the enamel: visible or detectable defects of the enamel; if enamel caries is in doubt, do not opt for it
2. Dentine caries ≤3 mm: the defect into the dentin does not exceed 3 mm in length and width measured with the periodontal probe PCP11
3. Dentine caries >3 mm: the defect into the dentin exceeds 3 mm in length and width measured with the periodontal probe PCP11
4. Filling: filled surfaces of teeth (without secondary caries) and crowns
5. Secondary caries: visible or detectable caries at the margin of fillings
6. Missing: all missing teeth except third molars
7. Others: missing anterior teeth due to trauma, missing premolars due to orthodontic treatment, crowns due to trauma (probands were ask for the reason of tooth loss), persistent teeth of the first dentition

This was the basis for the calculation of the DMFS index to characterize the SHIP sample in Tables 1 and 2, and to analyze the data using four ordered outcome levels on tooth level as described in more detail in the statistical analyses section.

**Table 1:** Demographic characteristics of participants aged 20–39 years of the Study of Health in Pomerania (SHIP), 1997–2001, *n* = 1210 Demographische Merkmale der Probanden im Alter von 20–39 Jahren der “Study of Health in Pomerania” (SHIP), 1997–2001, *n* = 1210

Variable	*n*	DMFS (half mouth)	*n*	Plaque (%),* n* = 1206
		Median (IQR)		Median (IQR)
Age group				
20–24 years	255	6 (4–8)	254	33 (17–63)
25–29 years	305	7 (5–9)	305	38 (17–63)
30–34 years	333	8 (6–10)	331	40 (20–67)
35–39 years	317	8 (6–10)	316	42 (25–67)
Gender				
Men	573	7 (5–9)	572	42 (21–67)
Women	637	8 (6–10)	634	38 (17–63)
School education				
<10 years	95	7 (5–9)	94	55 (30–80)
10 years	826	8 (6–10)	825	42 (21–67)
>10 years	289	7 (4–9)	287	29 (8–50)
Marital status				
Married	518	8 (6–10)	517	42 (21–63)
Married, living separately	20	9 (6–11)	20	50 (18–75)
Single	614	7 (5–9)	612	36 (17–63)
Divorced	55	8 (6–10)	54	46 (29–65)
Widowed	3	2 (0–4)	3	33 (29–67)
Household income (€/month)				
≤475	224	7 (5–9)	224	45 (21–69)
475<x≤700	237	7 (5–9)	235	40 (17–67)
700<x≤950	211	7 (5–9)	211	42 (17–63)
950<x≤1,250	264	8 (6–10)	262	42 (21–63)
>1,250	235	8 (6–9)	235	33 (15–58)
Smoking				
Never	376	5 (9–4)	374	33 (15–58)
Ex, <1 cig./day	142	7 (6–9)	142	33 (17–60)
Ex, 1–14 cig./day	64	7 (5–10)	64	29 (8–52)
Ex, ≥15 cig./day	85	7 (5–9)	85	42 (25–69)
Current, <1 cig./day	80	7 (5–9)	80	35 (17–54)
Current, 1–14 cig./day	214	7 (5–9)	214	42 (21–67)
Current, ≥15 cig./day	247	8 (5–10)	245	50 (29–75)
Orthodontic treatment				
Never	837	7 (6–9)	834	40 (20–63)
Currently	4	7 (5–8)	4	10 (4–25)
Formerly	360	7 (5–9)	359	38 (17–63)

*DMFT* Decayed-Missing-Filled Teeth index, *Ex* Former smoker, number of cigarettes (cig.) per day, *IQR* interquartile range

**Table 2:** Decayed-Missing-Filled Teeth index (DMFT) and plaque according to malocclusion variables of participants aged 20–39 years of the Study of Health in Pomerania (SHIP), 1997–2001, *n* = 1210 DMFT(„Decayed-Missing-Filled“)-Index und Plaque bezüglich Malokklusionsvariablen der Probanden im Alter von 20–39 Jahren der „Study of Health in Pomerania“ (SHIP), 1997–2001, *n* = 1210

Variable	*n*	DMFT (half mouth)	*n*	Plaque (%), *n* = 1206
		Median (IQR)		Median (IQR)
*Space conditions in the anterior region*				
Anterior crowding, upper arch lack of space				
No anterior crowding	643	8 (5–10)	639	42 (20–65)
≤½ lateral incisor width	483	7 (5–9)	483	38 (17–63)
½ <x≤ 1 lateral incisor width	68	6 (5–8)	68	38 (21–71)
>1 lateral incisor width	6	6 (4–8)	6	33 (29–38)
Anterior crowding, lower arch lack of space				
No anterior crowding	444	8 (6–10)	442	42 (17–63)
≤½ lateral incisor width	628	7 (5–9)	626	38 (17–63)
½ <x≤ 1 lateral incisor width	129	8 (6–9)	129	46 (20–75)
>1 lateral incisor width	9	8 (6–9)	9	33 (29–42)
Ectopic canine 13				
No	1089	7 (5–9)	1085	38 (17–63)
Yes	120	7 (5–9)	120	42 (21–70)
Ectopic canine 23				
No	1083	7 (5–9)	1079	38 (17–63)
Yes	127	7 (4–9)	127	38 (21–63)
Ectopic canine 33				
No	1108	7 (5–9)	1104	38 (17–63)
Yes	102	7 (5–9)	102	42 (25–70)
Ectopic canine 43				
No	1090	7 (5–9)	1086	38 (17–63)
Yes	120	7 (6–9)	120	42 (18–75)
Anterior spacing upper arch				
No	1056	8 (5–10)	1053	38 (17–63)
Yes	154	7 (5–8)	153	38 (20–63)
Anterior spacing lower arch				
No	1110	7 (5–9)	1106	38 (17–63)
Yes	100	8 (6–10)	100	42 (20–64)
*Space conditions in the posterior region*				
Posterior crowding right upper jaw				
No	958	7 (5–9)	954	38 (17–63)
Yes	252	7 (5–9)	252	38 (21–58)
Posterior crowding left upper jaw				
No	972	7 (5–10)	968	40 (17–63)
Yes	236	7 (5–9)	236	38 (21–58)
Posterior crowding left lower jaw				
No	898	8 (5–10)	894	40 (17–67)
Yes	312	7 (5–9)	312	38 (21–58)
Posterior crowding right lower jaw				
No	914	7 (5–9)	911	38 (17–65)
Yes	296	7 (5–9)	295	40 (25–60)
Posterior spacing right upper jaw				
No	1175	7 (5–9)	1171	38 (17–63)
Yes	35	6 (4–9)	35	30 (10–58)
Posterior spacing left upper jaw				
No	1168	7 (5–9)	1164	38 (18–63)
Yes	42	6 (4–8)	42	30 (8–50)
Posterior spacing left lower jaw				
No	1166	7 (5–9)	1162	38 (17–63)
Yes	44	8 (6–10)	44	40 (25–63)
Posterior spacing right lower jaw				
No	1160	7 (5–9)	1156	38 (17–63)
Yes	50	7 (5–9)	50	39 (21–60)
*Vertical overbite*				
Anterior open bite				
No	1165	7 (5–9)	1161	38 (17–63)
≤3 mm	37	7 (6–9)	37	38 (21–63)
>3 mm	8	10 (8–11)	8	85 (65–97)
Anterior edge to edge bite				
No	1132	7 (5–9)	1128	38 (17–63)
Yes	78	7 (6–9)	78	42 (21–69)
Deep anterior overbite				
No	912	7 (5–9)	909	38 (17–63)
Without gingival contact	211	7 (6–9)	210	39 (21–63)
With gingival contact	87	8 (5–10)	87	38 (17–63)
*Sagittal overjet*				
Retroclination/inversion of the upper incisors				
No	827	7 (5–9)	824	42 (21–67)
Yes	381	7 (5–9)	380	33 (17–58)
Anterior crossbite				
No	1150	7 (5–9)	1146	38 (17–63)
Yes	60	8 (5–9)	60	40 (29–65)
Negative overjet				
No	1196	7 (5–9)	1192	38 (17–63)
Yes	14	8 (6–9)	14	46 (29–67)
Increased sagittal overjet				
<4 mm	807	7 (5–9)	803	40 (17–65)
4–6 mm	304	8 (5–10)	304	35 (20–63)
>6 mm	97	7 (6–9)	97	42 (17–70)
*Lateral malocclusions*				
Left lateral crossbite				
No	1036	7 (5–9)	1033	38 (17–63)
Yes	174	8 (6–10)	173	42 (25–65)
Right lateral crossbite				
No	1035	7 (5–9)	1031	38 (17–63)
Yes	175	8 (6–10)	175	46 (25–71)
Left buccal nonocclusion				
No	1180	7 (5–9)	1176	38 (20–63)
Yes	30	6 (3–8)	30	23 (8–50)
Right buccal nonocclusion				
No	1181	7 (5–9)	1177	38 (20–63)
Yes	29	8 (5–9)	29	25 (8–54)
Left lateral open bite				
No	1198	7 (5–9)	1194	38 (17–63)
≤3 mm	11	6 (5–9)	11	46 (17–63)
>3 mm	1	10 (10–10)	1	70 (70–70)
Right lateral open bite				
No	1198	7 (5–9)	1195	38 (17–63)
≤3 mm	11	7 (5–9)	10	38 (25–63)
>3 mm	1	10 (10–10)	1	70 (70–70)
Left lateral edge to edge bite				
No	1023	7 (5–9)	1019	38 (17–63)
Yes	187	8 (5–10)	187	46 (21–67)
Right lateral edge to edge bite				
No	1018	7 (5–9)	1015	38 (17–63)
Yes	192	8 (6–10)	191	46 (21–71)
*Sagittal intermaxillary relationship in the canine region*				
Occlusion status left canine area				
Neutral	713	7 (5–9)	709	40 (17–65)
Distal ½ premolar width	276	8 (5–10)	276	38 (17–63)
Distal 1 premolar width	152	7 (5–9)	152	36 (17–58)
Mesial	69	7 (6–9)	69	50 (29–71)
Occlusion status right canine area				
Neutral	747	7 (5–9)	743	38 (17–63)
Distal ½ premolar width	246	7 (5–10)	246	38 (20–63)
Distal 1 premolar width	139	8 (6–10)	139	33 (15–55)
Mesial	78	7 (6–10)	78	59 (33–79)
Asymmetry				
Symmetry	713	7 (5–9)	709	38 (17–63)
Neutral and distal ½	230	8 (5–10)	230	42 (17–63)
Neutral and distal 1	94	8 (5–9)	94	33 (15–50)
Neutral and mesial	64	8 (6–10)	64	53 (29–84)
Distal ½ and distal 1	76	7 (5–10)	76	33 (17–55)
Distal ½ and mesial	22	7 (5–10)	22	63 (33–75)
Distal 1 and mesial	11	7 (6–8)	11	63 (29–75)

*IQR* interquartile range

Visual inspection and probing with the dental probe PCP11 determined the presence or absence of plaque and calculus on test teeth 1, 3, and 6 in the selected quadrants, and the proportion of sites with plaque was calculated per participant. If a test tooth was missing, the distal adjacent tooth was examined instead. Each of these teeth was scored at four sites: distobuccal, midbuccal, mesiobuccal, midlingual.

### Quality control

Eight experienced and calibrated dentists performed the dental examinations. Training of examiners and consensus discussions were carried out before the study started and training/calibration sessions were repeated twice yearly while the study was ongoing. Orthodontic calibration of the examiners was based on the examination of 30 pairs of casts showing complex symptoms of malocclusion, examination was repeated after several days. Intra- and interexaminer agreement were measured by Cohen’s kappa (κ) [25, 26]. Cohen’s κ values ranged from 0.66–0.81, meaning “good agreement” [41]. The calibration exercises for the caries scores consisted of each examiner performing two examinations on each of 10 and 5 test participants one to two weeks apart. Examiners applied the eight categories for caries as described in the manual for SHIP‑0. On surface level, which was the basis for calibration and certification, very good Cohen’s κ values were reached for intra- and interexaminer reliability (0.9–1.0 and 0.93–0.96, respectively [26, 39]). On the tooth level as used herein, good κ values were reached for intra- and interexaminer reliability (0.69–1.0 and 0.70–1.0, respectively).

### Statistical analyses

To avoid selection bias, subject’s age range was restricted to 20–39 years; older subjects have a higher proportion of missing orthodontic variables due to missing teeth. As shown for the relationship between malocclusion and periodontal disease [4], confounding by tooth type across jaws required modelling on subject, jaw, and tooth levels. As is common in multilevel analyses [16], the outcome (caries) is measured on the tooth level, whereas some covariates are at the subject level, for example gender, and other covariates are at the tooth level, including all malocclusion variables except distal and mesial occlusion [4]. Thus, the 33 malocclusion variables on the subject level were transformed into 18 corresponding variables on the tooth level [4]. Thus, ectopic canines on the tooth level could occur only at 13, 23, 33, or 43 [4]. For crowding (and spacing as well), a single variable instead of two variables for anterior and posterior regions may be desirable. We addressed this coding scheme only in sensitivity analyses because the six joint tests for the global malocclusion conditions, including space conditions in the anterior region and lateral malocclusions, were clearly of clinical and statistical interest. Moreover, crowding was assessed differently in the anterior and posterior regions. The malocclusion variables were simultaneously fitted in ordinal logistic multilevel models using the “meologit” procedure (Stata software, release 14.2; Stata Corporation, College Station, TX, USA). The four ordered outcome levels were (1) sound, (2) carious defects into the enamel, (3) caries (dentine caries ≤3 mm, dentine caries >3 mm, filling, or secondary caries), and (4) tooth loss. Because pitfalls of ignoring the hierarchy in dental research (subject, tooth, surface; subject, jaw, tooth) have been well-known for 20 years [17], multilevel models have been widely used for answering complex research questions, especially when the tooth type is a confounder on a level different from the subject level [4, 18]. Herein, the three hierarchical levels subject, jaw, and tooth were included as random effects [36]; age, gender, school education (3 levels in accordance with the former east German school system), marital status (5 categories), jaw, tooth type (7 levels), the interaction between jaw and tooth type [21], and monthly household equivalence income (1 € = 1.956 German marks) were included as fixed effects [30]. Restricted cubic splines with three knots were used to allow for departures from linearity for age and income. Income was considered only in additional analyses because, unlike school education, it was linked with adulthood rather than childhood and, therefore, not assumed to be a confounder. As orthodontic treatment is part of the effect to be studied, it was not included into the model because “a confounder must not be an effect of the exposure” [37]. Odds ratios (OR) with 95% confidence intervals (CI) and *p*-values are provided. For any cut point of the outcome on four levels, ORs in ordinal logistic regression models can be interpreted as those in binary logistic regression models; note that the ordinal logistic regression model has fewer assumptions than the ordinary least squares regression model [22].

## Results

**Fig. 1:**
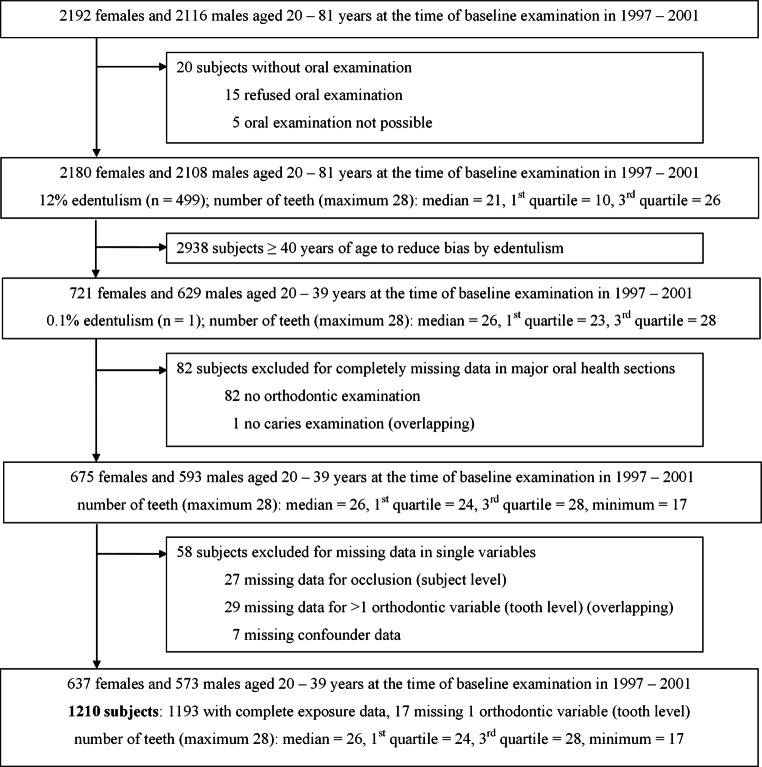
Flow chart the sample of Study of Health in Pomerania (SHIP), a population-based study in northeastern Germany, 1997–2001: Displayed are all excluded subjects due to the selected age stratum and missing variables Flussdiagramm der Stichprobe von “Study of Health in Pomerania” (SHIP), einer bevölkerungsbezogenen Studie in Nordostdeutschland, 1997–2001: Dargestellt sind alle aufgrund der Altersbegrenzung und fehlender Variablen ausgeschlossenen Probanden

The analysis sample consisted of 1210 participants with a median age of 30 years (interquartile range [IQR] 25–35 years). Of these patients, 30% had previously undergone orthodontic treatment. Four patients (<0.5%) were under treatment at the time of examination. (Fig. 1). The median DMFT half mouth was 7 (IQR 5–9 teeth). Participants’ general characteristics according to caries (DMFT) and plaque are shown in Table 1. Notably, the difference in plaque was very small comparing *never* and *former orthodontic treatment* (median: 40 and 38%, respectively). The orthodontic characteristics are shown in Table 2. The most common malocclusion was anterior crowding of the lower jaw in 766 of the 1210 subjects. Lateral open bite was observed in 12 subjects and was the least common malocclusion. According to intermaxillary relationships in the canine area, 44.3% of the subjects showed a neutral occlusion on both sides. Table 3 displays malocclusion in relation to orthodontic treatment for nontreated participants and participants who had previously undergone orthodontic treatment.

**Table 3:** Malocclusion and orthodontic treatment in participants aged 20–39 years of the Study of Health in Pomerania (SHIP), 1997–2001, *n* = 1187 (4 subjects with current treatment, 9 missing treatment values) Malokklusionen und kieferorthopädische Behandlung der Probanden im Alter von 20–39 Jahren der “Study of Health in Pomerania” (SHIP), 1997–2001, *n* = 1187 (4 Probanden mit aktueller Behandlung, 9 fehlende Behandlungswerte)

Variable	No (*n* = 837)		Formerly (*n* = 360)	
	*n*	%	*n*	%
*Space conditions in the anterior region*				
Anterior crowding, upper arch lack of space				
No anterior crowding	478	57.7	158	44.1
≤½ lateral incisor width	311	37.5	167	46.6
½ <x≤ 1 lateral incisor width	37	4.5	31	8.7
>1 lateral incisor width	3	0.4	2	0.6
Anterior crowding, lower arch lack of space				
No anterior crowding	333	39.8	107	29.7
≤½ lateral incisor width	425	50.8	194	53.9
½ <x≤ 1 lateral incisor width	73	8.7	56	15.6
>1 lateral incisor width	6	0.7	3	0.8
Ectopic canine 13				
No	760	90.9	316	87.8
Yes	76	9.1	44	12.2
Ectopic canine 23				
No	761	90.1	309	85.8
Yes	76	9.1	51	14.2
Ectopic canine 33				
No	779	93.1	317	88.1
Yes	58	6.9	43	11.9
Ectopic canine 43				
No	779	93.1	298	82.8
Yes	58	6.9	62	17.2
Anterior spacing upper arch				
No	726	86.7	321	89.2
Yes	111	13.3	39	10.8
Anterior spacing lower arch				
No	759	90.7	339	94.2
Yes	78	9.3	21	5.8
*Space conditions in the posterior region*				
Posterior crowding right upper jaw				
No	681	81.4	267	74.2
Yes	156	18.6	93	25.8
Posterior crowding left upper jaw				
No	681	81.5	280	80.0
Yes	155	18.5	79	22.0
Posterior crowding left lower jaw				
No	644	76.9	244	67.8
Yes	193	23.1	116	32.2
Posterior crowding right lower jaw				
No	653	78.0	250	69.4
Yes	184	22.0	110	30.6
Posterior spacing right upper jaw				
No	812	97.0	350	97.2
Yes	25	3.0	10	2.8
Posterior spacing left upper jaw				
No	806	96.3	349	96.9
Yes	31	3.7	11	3.1
Posterior spacing left lower jaw				
No	806	96.3	347	96.4
Yes	31	3.7	13	3.6
Posterior spacing right lower jaw				
No	803	95.9	345	95.8
Yes	34	4.1	15	4.2
*Vertical overbite*				
Anterior open bite				
No	815	97.4	338	93.9
≤3 mm	19	2.3	17	4.7
>3 mm	3	0.4	5	1.4
Anterior edge to edge bite				
No	786	93.9	335	93.1
Yes	51	6.1	25	6.9
Deep anterior overbite				
No	635	75.9	267	74.2
Without gingival contact	141	16.8	68	18.9
With gingival contact	61	7.3	25	6.9
*Sagittal overjet*				
Retroclination/inversion of the upper incisors				
No	566	67.8	254	70.6
Yes	269	32.2	106	29.4
Anterior crossbite				
No	805	96.2	333	92.5
Yes	32	3.8	27	7.5
Negative overjet				
No	831	99.3	352	97.8
Yes	6	0.7	8	2.2
Increased sagittal overjet				
<4 mm	571	68.3	228	63.5
4–6 mm	212	25.4	89	24.8
>6 mm	53	6.3	42	11.7
*Lateral malocclusions*				
Left lateral crossbite				
No	727	86.9	298	82.8
Yes	110	13.1	62	17.2
Right lateral crossbite				
No	725	86.6	300	83.3
Yes	112	13.4	60	16.7
Left buccal nonocclusion				
No	816	97.5	351	97.5
Yes	21	2.5	9	2.5
Right buccal nonocclusion				
No	815	97.4	353	98.1
Yes	22	2.6	7	1.9
Left lateral open bite				
No	832	99.4	355	98.6
≤3 mm	5	0.6	4	1.1
>3 mm	0	0.0	1	0.3
Right lateral open bite				
No	830	99.2	355	98.6
≤3 mm	7	0.8	4	1.1
>3 mm	0	0	1	0.3
Left lateral edge to edge bite				
No	708	84.6	305	84.7
Yes	129	15.4	55	15.3
Right lateral edge to edge bite				
No	710	84.8	299	83.1
Yes	127	15.2	61	16.9
*Sagittal intermaxillary relationship in the canine region*				
Occlusion status left canine area				
Neutral	500	59.7	203	56.4
Distal ½ premolar width	185	22.1	90	25.0
Distal 1 premolar width	107	12.8	44	12.2
Mesial	45	5.4	23	6.4
Occlusion status right canine area				
Neutral	522	62.4	216	60.0
Distal ½ premolar width	167	20.0	76	21.1
Distal 1 premolar width	101	12.1	37	10.3
Mesial	47	5.6	31	8.6
Asymmetry				
Symmetry	483	57.7	221	61.4
Neutral and distal ½	161	19.2	68	18.9
Neutral and distal 1	77	9.2	16	4.4
Neutral and mesial	38	4.5	25	6.9
Distal ½ and distal 1	52	6.2	23	6.4
Distal ½ and mesial	19	2.3	3	0.8
Distal 1 and mesial	7	0.8	4	1.1

**Fig. 2:**
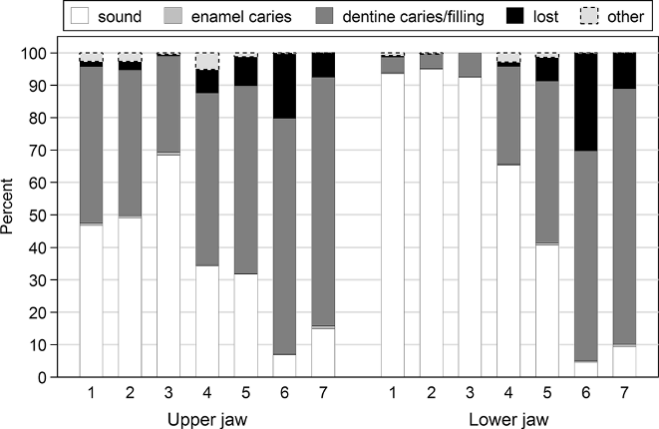
Stacked bar chart of sound, enamel caries, dentine carious/filled/secondary caries, and missing teeth according to tooth type and jaw (half mouth). “Other” includes missing due to trauma or due to orthodontic extraction. Differences between jaws regarding sound teeth justify the level “jaw” in the analysis Balkendiagramm für gesunde Zähne, Zähne mit Schmelzkaries, kariöse und gefüllte Zähne, Zähne mit sekundärer Karies sowie fehlende Zähne geordnet nach Zahntyp und Kiefer (halbseitig). “Sonstige” umfasst das Fehlen von Zähnen aufgrund von Traumata oder kieferorthopädischen Extraktionen. Unterschiede zwischen Ober- und Unterkiefer in Bezug auf gesunde Zähne rechtfertigen die Ebene “Kiefer” in der Analyse

On the tooth level, out of the 16,675 teeth half mouth, 1196 teeth were missing, 7521 displayed caries into the dentin, and 72 revealed clinically detectable enamel caries lesions (7.2, 45.1, and 0.4%, respectively, Fig. 2; Table 4). Caries differs considerably by tooth type and jaw, especially for incisors and canines (Fig. 2).

**Table 4:** Caries (four ordered levels: sound, enamel caries, caries, tooth loss): ordinal multilevel model on 1210 subjects, 2420 jaws, and 16,675 teeth (4727 incisors, 2410 canines, and 9538 premolars and molars); odds ratios (OR) on tooth level are adjusted for age, gender, school education, marital status, jaw, tooth type, and the interaction between jaw and tooth type, and for the subject and jaw level Karies (4 geordnete Ebenen: gesund, Schmelzkaries, Karies, Zahnverlust): ordinales Mehrebenenmodell bei 1210 Probanden, 2420 Kiefern und 16.675 Zähnen (4727 Schneidezähne, 2410 Eckzähne und 9538 Prämolaren und Molaren); Chancenverhältnisse (Odds Ratios, OR) auf Zahnebene wurden an Alter, Geschlecht, Schulbildung, Familienstand, Kiefer, Zahntyp und die Wechselwirkung zwischen Kiefer und Zahntyp sowie an Personen- und Kieferebene angepasst

Variable	Teeth	Caries	Relative effect measure	Related test
	Frequency	Frequencies for enamel caries; caries; tooth loss	OR (95% CI)	*P* value (*P*_trend_)
*Space conditions in the anterior region*	–	–	–	*<0.0001*
Anterior crowding, lack of space	–	–	–	(0.0350)
No anterior crowding	14,055	61; 6915; 1190	1 (reference)	–
≤½ lateral incisor width	2202	11; 531; 6	0.84 (0.68–1.03)	0.0958
½ <x≤ 1 lateral incisor width	388	0; 69; 0	0.68 (0.45–1.03)	0.0660
>1 lateral incisor width	30	0; 6; 0	0.63 (0.18–2.26)	0.4824
Ectopic canines	214	3; 35; 0	1.25 (0.8–1.95)	0.3229
Anterior spacing	493	3; 85; 5	0.24 (0.17–0.33)	<0.0001
*Space conditions in the posterior region*	–	–	–	*<0.0001*
Posterior crowding	2675	9; 1418; 115	0.57 (0.49–0.66)	<0.0001
Posterior spacing	444	0; 197; 57	0.69 (0.5–0.95)	0.0230
*Vertical overbite*	–	–	–	*0.0412*
Anterior open bite	–	–	–	(0.0073)
No	16,507	72; 7461; 1193	1 (reference)	–
≤3 mm	136	0; 46; 2	2.08 (1.19–3.61)	0.0096
>3 mm	32	0; 14; 1	2.19 (0.74–6.51)	0.1582
Anterior edge to edge bite	307	3; 74; 6	0.90 (0.60–1.35)	0.6272
Deep anterior overbite	–	–	–	(0.0441)
No	15,519	64; 7207; 1176	1 (reference)	–
Without gingival contact	819	7; 217; 15	1.23 (0.95–1.60)	0.1179
With gingival contact	337	1; 97; 5	1.39 (0.95–2.04)	0.0888
*Sagittal overjet*	–	–	–	*0.0325*
Retroclination upper incisors	734	5; 353; 11	0.91 (0.71–1.16)	0.4492
Anterior crossbite	297	3; 67; 4	1.05 (0.66–1.69)	0.8249
Negative overjet	54	0; 18; 2	2.17 (0.84–5.62)	0.1107
Increased sagittal overjet	–	–	–	(0.0090)
No	15,114	66; 7073; 1171	1 (reference)	–
4–6 mm	1.182	5; 338; 17	1.31 (1.05–1.64)	0.0191
>6 mm	379	1; 110; 8	1.45 (1.00–2.11)	0.0517
*Lateral malocclusions*	–	–	–	*0.0051*
Lateral crossbite	1670	13; 891; 202	1.16 (0.94–1.43)	0.1742
Buccal nonocclusion	158	0; 82; 6	0.54 (0.33–0.87)	0.0116
Lateral open bite	–	–	–	(0.1119)
No	16,559	71; 7457; 1183	1 (reference)	–
≤3 mm	106	1; 58; 11	1.61 (0.77–3.39)	0.2085
>3 mm	10	0; 6; 2	3.47 (0.34–35.3)	0.2932
Lateral edge to edge bite	1885	11; 997; 238	1.21 (0.99–1.47)	0.0624
*Sagittal intermaxillary relationship in the canine region*	–	–	–	*0.0200*
Distal occlusion	–	–	–	(0.0047)
Neutral or mesial occlusion	8626	53; 3813; 556	1 (reference)	–
Distal ½ premolar width	4822	14; 2207; 391	1.27 (1.05–1.53)	0.0125
Distal 1 premolar width	3227	5; 1501; 249	1.31 (1.06–1.63)	0.0143
Mesial occlusion	1682	14; 752; 145	1.18 (0.90–1.55)	0.2245

For frequencies, each category is presented only if the variable (not the subgroup) has more than two levels. For variables on two levels, the frequency of the designated malocclusion category is presented; the frequency of the remaining category can be calculated (16,675 − frequency of the designated malocclusion category)

Likewise, the frequency of the caries level “sound” can be calculated (sound = tooth frequency − (enamel caries + caries + tooth loss), for example, 5889 = 14,055 − (61 + 6915 + 1190) in the first row)

*95% CI* 95% confidence interval

### Caries model

On the tooth level, the following malocclusions were associated with an increased odds ratio for caries, or more exactly, for tooth loss versus no tooth loos; or tooth loss or caries versus no caries; or tooth loss, caries, or enamel caries versus sound (Table 4): anterior open bite ≤3 mm (OR = 2.08, CI: 1.19–3.61, frequency among all incisors 2.9%) and increased sagittal overjet of 4–6 mm (OR = 1.31, CI: 1.05–1.64, frequency among all incisors 25.0%). Increased sagittal overjet of >6 mm (OR = 1.45, CI: 1.00–2.11, frequency among all incisors 8%) displayed a *p*-value of <0.1. Distal occlusion according to the sagittal intermaxillary relation in the canine region also displayed higher odds for caries with distal ½ premolar width (OR = 1.27, CI: 1.05–1.53, frequency among all teeth 28.9%) and distal 1 premolar width (OR = 1.31, CI: 1.06–1.63, frequency among all teeth 19.4%). For negative overjet, the data are consistent with a true OR between 0.84 and 5.62 (frequency among all incisors 1.1%). Some malocclusions were associated with a significantly reduced odds for caries: anterior spacing (OR = 0.24 CI: 0.17–0.33, frequency among all incisors: 10.4%), posterior spacing, (OR = 0.69 CI: 0.50–0.95, frequency among all posterior teeth 4.7%), posterior crowding (OR = 0.57 CI: 0.49–0.66 frequency among all posterior teeth 28.0%) and buccal nonocclusion (OR = 0.54 CI: 0.33–0.87, frequency among all posterior teeth: 1.7%), (Table 4).

Joint effects occurred for space conditions in the anterior region (*p* < 0.0001 for the global test with 5 degrees of freedom; Table 4), space conditions in the posterior region (*p* < 0.0001), vertical overbite (*p* = 0.0412), sagittal overjet (*p* = 0.0325), lateral malocclusions (*p* = 0.0051), and sagittal intermaxillary relationship in the canine region (*p* = 0.0200). The joint effect for increased sagittal overjet and distal occlusion, which were correlated, was statistically significant (*p* = 0.0011 for the global test with 4 degrees of freedom).

### Sensitivity analyses using a single variable for crowding and spacing, respectively

Whereas anterior and posterior spacing can be combined into a single spacing variable in a natural way, posterior crowding can be combined with different levels of anterior crowding. Counting posterior crowding as the lowest level of the presence of anterior crowding, the ORs were 0.65 (95% CI: 0.58–0.74; *p* < 0.0001), 0.64 (95% CI: 0.43–0.95; *p* < 0.0255), and 0.60 (95% CI: 0.17–2.14; *p* = 0.4348) from the lowest to the highest crowding level, respectively. The OR of spacing was 0.38 (95% CI: 0.30–0.48; *p* < 0.0001). Counting posterior crowding as the middle level of anterior crowding, the OR of the middle level was 0.56 (95% CI: 0.49–0.65; *p* < 0.0001). Of note, the 95% CIs for anterior and posterior spacing did not overlap in the main analysis (Table 4).

### Sensitivity analyses including household income

Including household income did not lead to a change >10% in the ORs of malocclusion variables in the reduced sample of 1171 subjects.

## Discussion

Capitalizing on a large sample size from the general population, this is the first study to investigate the association between malocclusions and caries on tooth, jaw and subject levels in adults in a single model. The benefit of orthodontic treatment on oral health including caries prevention is a matter of ongoing debate in the literature as well as in political demands for scientific proof [2, 5, 8, 12]. The extensive dataset of SHIP enables analyses with multilevel models that consider the nested character of the data (tooth level under consideration of the jaw and subject level) [36]. Such extensive analyses including all forms of malocclusion have not been possible in the past.

Although a marked decline in caries has been noticed during the last 30 years in Western countries, caries still represents a relevant dental problem [29, 38, 39]. DMFT values of our subsample are not comparable to other population-based surveys due to the selection criteria described above. Caries prevalence of the sample from SHIP, which has been published previously, is higher compared to other nationwide data from Western European countries in the same decade [27, 31, 38, 39]. Higher numbers of filled and missing teeth in seniors compared to Swedish and US surveys may be based on limited caries prevention programs or unavailability of fluoridated tooth paste before 1989 [39]. DMFT values in the comparable age group of the 35–44 year olds are slightly elevated compared to a German nationwide survey, which was conducted in 2005 [39, 43]. The Fourth German Health Study also reported elevated values for the former East Germany [43].

Beside socioeconomic or cohort effects, several local factors such as improper tooth alignment have also been connected to an increased caries prevalence [1]. Although policy makers have long demanded for a causal relationship between different forms of malocclusion and caries, these associations have been only insufficiently investigated [1, 7].

Our analyses resulted in a heterogeneous picture with some positive and also inverse associations between malocclusion and caries which have not been investigated in detail before. We observed positive associations for caries and increased sagittal overjet, anterior open bite and distal occlusion. These associations have not been reported previously in adult samples of epidemiologic surveys. In spite of statistical significance, the strength of the association remained moderate. Just anterior open bite up to 3 mm displayed an OR of 2. It occurred, however, in only 0.8% of the relevant teeth. In adolescents, however, this association was previously reported. Reduced salivary flow and a mouth breathing habit may have enhanced susceptibility to dental caries [33]. In one of the few studies on adults that also included maxillary overjet, Helm and Petersen did not find associations of any malocclusion variable with caries incidence [23]. In pediatric epidemiological samples, an association to increased overjet and open bite could be established at least for the mixed dentition [33, 40]. Whereas the study by Stahl and Grabowski displayed that mandibular overjet was associated with higher caries incidence, high plaque scores were found in 12-year-old children with extreme maxillary overjet. The authors assumed a more difficult tooth cleaning and prolonged plaque accumulation in these cases that might lead to higher caries values [11]. In a study by Feldens et al. on 509 Brazilian adolescents, higher caries scores were associated with handicapping malocclusion, maxillary irregularity and abnormal molar relationships. The authors also speculated that prolonged biofilm formation might have increased the caries risk [13].

Some studies that found an association between malocclusion and caries did not distinguish between malocclusion traits but used sum scores or indices [7, 14, 15], whereas several other studies did not confirm an increased risk [10, 12, 44]. An aspect to recognize here is the age differences between the studied populations, i.e., caries had a longer course to develop in adult subjects with certain malocclusion traits compared to the pediatric and adolescent populations with the same traits, where mixed or permanent dentitions in the latter had shorter periods of exposure to caries-inducing factors.

Crowding of the anterior or posterior teeth was not associated with an increased caries score. Posterior crowding was even significantly associated with lower caries prevalence, a result which has also been observed previously [20]. Our results strengthen the assumption that despite the irregular tooth alignment and potential plaque accumulation, these factors do not necessarily lead to a higher caries rate [2, 23]. Our study followed the recommendations by Hafez et al. who did not confirm or refute a causal relationship between crowding and dental caries [20]. Until 2011, they found only eight reliable studies on that topic and claimed that well-controlled studies with larger sample sizes with standardized diagnostic tools would be necessary to resolve the question. Finally, the only plausible hypothesis on the link between malocclusion and caries that focus on plaque accumulation was also rejected in our large sample study.

We found inverse associations between caries and malocclusions as anterior and posterior spacing as well as posterior crowding and buccal nonocclusion, which were also observed in part by several studies in adolescents and adults [9, 20]. Anterior and posterior spacing within the context of caries risk is assumed to play a protective role, as plaque removal would be easier to achieve with the absence of proximal contacts [32].

Traumatic events leading to increased caries values might also occur in persons with malocclusion as for instance increased sagittal overjet [34]. To avoid this influence, the examination in SHIP 0 did not count traumatic events and tooth loss due to trauma or orthodontic tooth extractions as missing teeth in assessing the DMFS. However, the caries risk was increased in persons with an overjet of more than 6 mm compared to an overjet of 4–6 mm. Furthermore, because periodontal disease that finally leads to tooth loss has been linked with increased sagittal overjet [4], we chose our sample within an age range of 20 to 39 years, to reduce the risk of complete tooth loss due to periodontal breakdown. Additional analyses (not shown) of our data on Decayed Filled Teeth (DFT) level resulted in lower OR values but yielded the same tendencies.

Our study has several strengths as the large sample size provided adequate statistical power. The target population was limited to ages within a certain range, reducing the risk of bias due to tooth loss or missing values. We performed a standardized data collection with a high degree of quality management, including calibration and certification of caries examiners on surface level. Clinical experience is reflected by modelling jaw differences in tooth types, which is important for incisors and canines. Moreover, tooth type is a key confounder for the relationship between malocclusion and caries, which can be dealt with in multilevel models as used herein, but not in classical regression models, which ignore the hierarchically structured data [16]. It is this hierarchical structure that can model caries and tooth loss on an ordinal scale, whereas this natural ordering is lost by using the DMFT in subject level analysis. Thus, the severe information loss accompanied with choosing DMFT and subject level analysis ignores basic principles in statistics—it is far from being the best “for the money”.

Limitations are the cross-sectional analysis not allowing the establishment of causal relationships. A high prevalence of malocclusions was present in study participants who reported former orthodontic treatment. This was not unexpected since interviewing adult subjects about previous orthodontic treatments provides only orientational data. No information was given on orthodontic treatment length, applied methods and success rate [25].

## Conclusion

The current results and reviews from the literature suggest that associations between caries and malocclusion depend on the kind of malformation. Anterior open bite (OR = 2.08, CI: 1.19–3.61), increased sagittal overjet (OR = 1.31, CI: 1.05–1.64) and distal occlusion (OR = 1.31, CI: 1.06–1.63) were positively associated with caries, whereas spacing, posterior crowing and buccal nonocclusion were negatively associated. Caries and malocclusion, however, were not far reaching associated. Anterior crowding was not associated with caries nor displayed higher plaque scores compared to no crowding. Causality of the detected associations have to be examined in longitudinal analyses.
